# Antithrombotic Therapy After PCI in High-Risk Cardiovascular Patients: Navigating Complexity Beyond Guidelines

**DOI:** 10.3390/jcm15135110

**Published:** 2026-07-01

**Authors:** Francesco Antonio Veneziano, Leonardo De Luca

**Affiliations:** 1 Department of Cardiology, AULSS 6 Euganea, 35043 Padua, Italy; francescoantonio.veneziano@aulss6.veneto.it; 2 Division of Cardiology, Fondazione IRCCS Policlinico San Matteo, 27100 Pavia, Italy

**Keywords:** antithrombotic therapy, percutaneous coronary intervention, high bleeding risk, dual antiplatelet therapy, triple antithrombotic therapy, atrial fibrillation, chronic kidney disease, active cancer, frailty

## Abstract

Antithrombotic therapy is a cornerstone of contemporary cardiovascular management, substantially reducing thrombotic complications across a wide spectrum of cardiovascular conditions. However, the application of antithrombotic strategies in routine clinical practice remains challenging. Many patients commonly encountered in real-world settings—including elderly individuals, patients with multimorbidity, active malignancy, or advanced chronic kidney disease—are underrepresented in randomized clinical trials. Consequently, current guideline recommendations, although evidence-based, may not fully capture the complexity of these populations. In daily practice, clinicians are often required to balance competing risks of thrombosis and bleeding in patients characterized by multiple comorbidities, polypharmacy, and varying degrees of frailty, particularly when more than one indication for antithrombotic therapy coexists, such as atrial fibrillation in patients undergoing percutaneous coronary intervention. In this critical narrative review, we address key high-risk scenarios—frailty, advanced chronic kidney disease, active cancer, and atrial fibrillation in patients undergoing recent percutaneous coronary intervention—where standard antithrombotic strategies require special consideration. We critically appraise the limitations of existing bleeding risk scores and propose practical considerations for tailoring dual antiplatelet therapy duration, minimizing triple therapy, selecting antithrombotic combinations, and reassessing risk over time to optimize net clinical benefit.

## 1. Introduction

Antithrombotic therapy has become a cornerstone of modern cardiovascular management by attenuating a wide range of thrombotic and ischemic events in patients with coronary artery disease, particularly after percutaneous coronary intervention (PCI), while inevitably increasing the risk of bleeding. Consequently, achieving the right balance between ischemic benefit and bleeding risk is essential to optimize net clinical outcome and is therefore critical in modern cardiovascular practice [1,2]. The application of guideline-driven antithrombotic treatment in routine care has often proved problematic, because patients encountered in everyday practice usually differ from those who are included in clinical trials. Today, coronary patients are often older, clinically complex, affected by multiple comorbidities, and exposed to polypharmacy, which may influence both thrombotic risk and bleeding tendency simultaneously [3,4,5].

Current international guidelines provide a structured framework for the management of antithrombotic therapy across acute and chronic coronary syndromes, with dual antiplatelet (DAPT) therapy representing the standard treatment for most patients undergoing percutaneous coronary intervention (PCI) or presenting with acute coronary syndromes. Nevertheless, treatment intensity and duration should be individualized according to bleeding risk and overall clinical context, reflecting the growing recognition that a uniform therapeutic strategy may not be appropriate for all patients [1,2,6,7].

To facilitate risk stratification, several efforts have focused on the identification of patients at high bleeding risk. Consensus definitions such as the Academic Research Consortium High Bleeding Risk (ARC-HBR) criteria attempt to standardize bleeding risk assessment by integrating clinical variables, including advanced age, anemia, renal dysfunction, previous bleeding events, and other comorbid conditions associated with increased hemorrhagic vulnerability [8,9].

Beyond traditional cardiovascular risk factors, several conditions associated with biological vulnerability have emerged as important determinants of prognosis and therapeutic risk in patients with coronary artery disease. Geriatric syndromes such as frailty, as well as chronic comorbid conditions including chronic kidney disease and active malignancy, are increasingly prevalent in contemporary cardiovascular populations. These conditions often coexist with anemia, systemic inflammation, polypharmacy, and functional decline, contributing to a complex clinical profile characterized by both increased thrombotic susceptibility and heightened bleeding risk [3,10,11,12].

Importantly, these high-risk populations are markedly underrepresented in randomized trials; therefore, guideline recommendations frequently require careful clinical interpretation and adaptation.

This critical narrative review focuses on antithrombotic therapy after PCI in selected high-risk clinical scenarios, including frailty, chronic kidney disease, active cancer, and atrial fibrillation requiring oral anticoagulation. The aim is to discuss the limitations of current risk stratification tools and to provide practical considerations for tailoring antithrombotic intensity, treatment duration, aspirin discontinuation, triple therapy minimization, and longitudinal risk reassessment in routine clinical practice.

## 2. Methods and Scope of the Review

This article was conceived as a critical narrative review rather than a systematic review or meta-analysis. The manuscript focuses on antithrombotic therapy after percutaneous coronary intervention (PCI) in selected high-risk cardiovascular populations. Relevant evidence was identified through a targeted literature review of major international guidelines, consensus documents, randomized clinical trials, observational studies, meta-analyses, and clinically relevant review articles. The literature search was performed using PubMed/MEDLINE, with additional references identified from the bibliographies of selected articles. Search terms included combinations of “antithrombotic therapy”, “dual antiplatelet therapy”, “DAPT”, “PCI”, “high bleeding risk”, “ARC-HBR”, “PRECISE-DAPT”, “frailty”, “chronic kidney disease”, “active cancer”, “atrial fibrillation”, “oral anticoagulation”, and “triple therapy”. Frailty, chronic kidney disease, active cancer, and atrial fibrillation requiring oral anticoagulation after PCI were selected as the main clinical scenarios because they are common in contemporary cardiovascular practice, frequently involve overlapping thrombotic and bleeding risk, and remain insufficiently represented in randomized trials. The aim of the review was therefore not to provide an exhaustive summary of all antithrombotic indications, but to discuss selected scenarios in which guideline-based recommendations often require careful clinical interpretation and individualized application. Other antithrombotic settings, such as isolated venous thromboembolism, peripheral artery disease, valvular heart disease, transcatheter valve interventions, perioperative antithrombotic interruption, and advanced liver disease, were considered beyond the focused scope of the article.

## 3. Bleeding Risk and Ischemic Risk: The Central Trade-Off

The decision to prescribe antithrombotic therapy and determine its duration essentially requires balancing the reduction in ischemic events against the increased risk of bleeding. While antiplatelet agents and anticoagulants significantly reduce the incidence of myocardial infarction, stent thrombosis, and ischemic stroke, this gain also comes with the drawback of a greater risk of hemorrhage. Major bleeding can be catastrophic and has been associated with adverse clinical outcomes, premature cessation of antithrombotic treatment, and subsequent ischemic complications [13,14,15].

Contemporary guidelines advocate an individualized approach that weighs net clinical benefit by carefully balancing thrombotic and bleeding risks [1,2,6,7]. In practice, however, risk stratification remains difficult because ischemic and bleeding risk factors frequently coexist and share common determinants. Features that increase thrombotic risk often simultaneously heighten bleeding risk. As a result, aggressive regimens aimed at maximal ischemic protection may cause unacceptable bleeding, while more conservative strategies may fail to prevent thrombotic events. To assist clinicians in navigating this trade-off, multiple risk stratification tools have been developed.

Scores such as the DAPT score, PRECISE-DAPT, and the Academic Research Consortium High Bleeding Risk (ARC-HBR) criteria aim to identify patients at increased risk of hemorrhagic complications and guide the duration of dual antiplatelet therapy after PCI [16,17,18,19]. While these tools provide a useful framework for clinical decision-making, their predictive accuracy remains modest, and their applicability to complex real-world patients is often limited [20,21]. Importantly, most available scores provide a baseline estimate of risk but do not fully capture dynamic changes such as renal function deterioration, new anemia, active cancer treatment, thrombocytopenia, frailty progression, or the need for concomitant oral anticoagulation.

In recent years, increasing attention has been directed toward strategies aimed at mitigating bleeding risk while preserving ischemic protection. This interest partly reflects the recognition that currently available risk stratification tools provide only moderate discriminatory performance and may not fully capture the complexity of real-world patients. In this context, alternative antithrombotic approaches—including abbreviated durations of dual antiplatelet therapy after PCI—have been explored, although their optimal role in broader and more complex patient populations remains uncertain [22,23,24]. However, the selection of these strategies should be guided not only by baseline risk scores but also by clinical scenario, PCI complexity, concomitant indications for anticoagulation, and repeated reassessment over time.

## 4. Complex Clinical Scenarios in Antithrombotic Therapy

### 4.1. Frailty and Biological Age in Antithrombotic Decision-Making

Frailty is recognized as an important predictor of outcome and treatment sensitivity in cardiovascular disease. It is not merely an indicator of chronological age, but a multidimensional syndrome defined by a decrease in physiological reserve and an inability to adapt to homeostatic challenges. It reflects biological age and integrates several domains: performance and frailty testing (performance assessment of the musculoskeletal system), nutritional status, cognition, and number of morbid conditions [25,26]. In elderly patients with coronary artery disease, frailty is often characterized by sarcopenia, malnutrition, anemia, polypharmacy, and reduced functional capacity [3].

The prevalence of frailty is steadily increasing in contemporary cardiovascular populations, reflecting the progressive aging of patients presenting with coronary artery disease and acute coronary syndromes [26]. In this context, frailty has been consistently associated with worse clinical outcomes, including higher mortality, increased rehospitalization rates, and poorer functional recovery after cardiovascular interventions [3,27].

From a pathophysiological perspective, frail patients often present with multiple conditions that may simultaneously increase both thrombotic and bleeding vulnerability. Advanced age, chronic kidney disease, anemia, systemic inflammation, and polypharmacy contribute to alterations in vascular integrity, platelet function, and hemostatic balance. These factors may predispose frail individuals to hemorrhagic complications during antithrombotic therapy while also maintaining a substantial risk of recurrent ischemic events [28,29]. As a result, the management of antithrombotic therapy in this population requires careful clinical judgment and individualized therapeutic strategies.

Several observational studies have identified a clear link between frailty and adverse clinical outcomes among coronary revascularization patients. Pre-procedural frailty has been found to be a predictor of death and clinical events in elderly patients with stable coronary artery disease after undergoing PCI [30]. GRACE registry data also showed that frailty was an independent predictor of in-hospital bleeding after acute myocardial infarction, highlighting this population’s vulnerability to intensive antithrombotic regimens [31]. This finding has also been supported by another study showing that combining frailty scales with other bleeding risk assessments improves the prediction of hemorrhage in elderly patients with acute coronary syndromes (ACS) [32]. An evaluation of frailty and antithrombotic use has also been investigated using randomized trial data.

In the EXTEND-DAPT study, patients with claims-defined frailty had a significantly higher incidence of net adverse clinical events—including death, myocardial infarction, stroke, and major bleeding—compared with non-frail individuals after PCI [4].

These findings highlight the importance of routine frailty assessment when deciding the intensity and duration of dual antiplatelet therapy. In clinical practice, antithrombotic strategies in frail patients generally follow the same principles as in the general population, with adjustments based on bleeding risk. However, elderly and frail patients remain particularly prone to bleeding complications after coronary stenting, especially in the presence of multiple comorbidities and polypharmacy [33]. Shorter durations of dual antiplatelet therapy have been investigated in older patients undergoing PCI, with data suggesting reduced bleeding without a significant increase in ischemic events in selected populations [27,34]. Nevertheless, frail individuals continue to be markedly underrepresented in randomized trials, limiting the direct applicability of current guideline recommendations [1,6]. This evidence gap underscores the need for dedicated studies in frail and biologically vulnerable patients with coronary artery disease.

From a practical standpoint, frailty should prompt a more cautious and dynamic approach to antithrombotic therapy after PCI. Whenever feasible, clinicians should complement conventional bleeding risk scores with a structured assessment of biological vulnerability, including functional status, history of falls, anemia, renal function, cognitive impairment, nutritional status, polypharmacy, and treatment adherence [25,26]. In frail patients with high bleeding risk and no major ischemic features, abbreviated DAPT or early transition to a less intensive antithrombotic strategy may be considered, particularly when contemporary drug-eluting stents are used [32,33,34]. Conversely, in frail patients with acute coronary syndromes, complex PCI, prior stent thrombosis, or extensive coronary disease, ischemic risk may still justify a more intensive strategy if bleeding risk is closely monitored. Reassessment should be performed early after discharge and repeated at 1–3 months, or sooner in the event of bleeding, new anemia, falls, hospitalization, renal function deterioration, or relevant drug changes.

### 4.2. Patients with Chronic Kidney Disease

Chronic kidney disease (CKD) represents one of the most prevalent and clinically significant comorbidities seen in cardiovascular patients. Overall, 10–15% of the population suffers from renal dysfunction; however, this value increases considerably in patients with coronary artery disease and in patients presenting with acute coronary syndromes [35,36]. Generally, CKD is defined as a reduced estimated glomerular filtration rate (eGFR < 60 mL/min/1.73 m^2^) for at least 3 months and/or the presence of markers of kidney damage, such as albuminuria. The severity of renal dysfunction is usually categorized according to kidney disease: Improving Global Outcomes (KDIGO) staging, considering both eGFR value and albuminuria class, and is directly correlated with the incremental increase in cardiovascular risk [35].

From a cardiovascular perspective, CKD is associated with a markedly increased risk of both thrombotic and hemorrhagic complications, creating a complex and often paradoxical hemostatic profile. Patients with impaired renal function frequently present with accelerated atherosclerosis, endothelial dysfunction, chronic inflammation, and vascular calcification, all of which contribute to a heightened risk of ischemic cardiovascular events, including myocardial infarction, stroke, and peripheral arterial disease [37,38]. At the same time, CKD patients are also predisposed to bleeding complications, with observational studies demonstrating a significantly higher incidence of major hemorrhagic events compared with individuals with preserved renal function [39,40].

The pathophysiological mechanisms underlying this dual thrombotic and hemorrhagic tendency are multifactorial and involve complex alterations of platelet function, endothelial integrity, and the coagulation cascade. Uremic toxin accumulation, chronic inflammatory activation, oxidative stress, and endothelial dysfunction contribute to a prothrombotic environment characterized by increased tissue factor expression, platelet activation, and enhanced thrombin generation [35,37]. In parallel, several mechanisms promote bleeding vulnerability, including platelet dysfunction, impaired platelet–vessel wall interaction, anemia, and abnormalities in coagulation pathways related to the uremic milieu [41]. As a result, CKD patients often exhibit a fragile hemostatic equilibrium in which both thrombosis and bleeding risks are simultaneously amplified. These pathophysiological changes have important clinical implications for optimizing antithrombotic treatment.

The use of antiplatelet agents and anticoagulants in CKD remains particularly challenging due to increased bleeding risk and altered pharmacokinetics from reduced renal clearance [42,43]. Although antiplatelet therapy reduces myocardial infarction rates, it is associated with a substantial increase in major and minor bleeding [44]. Observational data show that patients with CKD on dual antiplatelet therapy after PCI experience both higher ischemic burden and greater bleeding complications, especially in more advanced stages of renal failure [45]. Importantly, patients with advanced CKD are markedly underrepresented in major antithrombotic trials. As a result, current guidelines, which are largely derived from patients with normal renal function, have limited applicability in this high-risk population [36].

Clinically, CKD should not be treated as a single risk category, but rather stratified according to both the severity and stability of renal dysfunction. In patients with moderate CKD, antiplatelet strategies after PCI may often follow standard recommendations, although DAPT duration and drug selection should be guided by the concomitant bleeding profile, anemia, ischemic risk, and PCI complexity [35,36,46]. In advanced CKD, and particularly in patients with severely reduced eGFR or dialysis-dependent kidney disease, the therapeutic balance becomes more precarious because anemia, uremic platelet dysfunction, drug accumulation, and fluctuating renal function may amplify bleeding vulnerability. Therefore, prolonged combination therapy should be avoided unless clearly justified by high ischemic or procedural risk. In patients requiring oral anticoagulation, renal function should be assessed using creatinine clearance or estimated glomerular filtration rate to guide DOAC eligibility and dose adjustment, while recognizing that evidence is more limited in severe CKD and end-stage kidney disease [42,46,47]. Real-world data in atrial fibrillation also suggest that renal dysfunction is associated with progressively higher thromboembolic, bleeding, and mortality risk, supporting repeated reassessment rather than a single baseline therapeutic decision [47]. Reassessment of renal function, hemoglobin, and bleeding symptoms should be performed periodically and whenever acute kidney injury, contrast exposure, hospitalization, infection, dehydration, cancer therapy, or new interacting drugs occur.

### 4.3. Patients with Cancer and Active Malignancy

Active malignancy is one of the most rapidly growing clinical scenarios faced in current cardiovascular practice. With improvements in oncologic therapy, cancer survivorship is increasing, leading to a growing number of patients living with both malignancy and cardiovascular disease. As a result, cardiologists increasingly face the challenge of managing antithrombotic therapy in patients with active malignancy who undergo PCI, have atrial fibrillation (AF), or develop venous thromboembolism (VTE) during oncologic treatment [48,49].

Malignancy profoundly disrupts the hemostatic system with a dynamic and tenuous balance between thrombotic and hemorrhagic events. The process of cancer itself inherently leads to a prothrombotic milieu via numerous mechanisms, including activation of coagulation factors by the tumor, inflammation, endothelial dysfunction, as well as release of procoagulant factors, including tissue factor and cancer-associated microparticles [50,51,52]. Moreover, the management of malignancy via the use of anticancer therapies (including chemotherapy, targeted agents, and immunotherapies) can also increase rates of endothelial damage and platelet activation [53].

At the same time, patients with cancer are also exposed to a substantial risk of bleeding. Tumor-related factors such as local tissue invasion, mucosal disruption, thrombocytopenia, and bone marrow suppression may significantly impair hemostasis. Furthermore, cancer-related comorbidities—including anemia, renal dysfunction, liver disease, and malnutrition—may further increase hemorrhagic vulnerability. Observational studies have shown that anticoagulant-related bleeding occurs in approximately 8–10% of patients with cancer-associated VTE during the first year of treatment, with gastrointestinal and genitourinary bleeding representing the most common sites [54,55].

The risk of bleeding also varies substantially according to tumor type and disease stage. Gastrointestinal, genitourinary, and intracranial malignancies are consistently associated with a higher incidence of hemorrhagic complications, particularly in patients receiving anticoagulant therapy [56]. In contrast, other malignancies such as breast cancer appear to be associated with relatively lower bleeding risk [55]. The presence of metastatic disease, systemic anticancer therapies, and corticosteroid exposure may further modify individual bleeding susceptibility [53,57].

These considerations complicate the management of antithrombotic therapy in cancer patients with cardiovascular disease. For example, patients with atrial fibrillation and active malignancy often require oral anticoagulation for stroke prevention while simultaneously facing a higher bleeding risk [58]. Meta-analyses suggest that direct oral anticoagulants (DOACs) may provide a more favorable safety profile compared with vitamin K antagonists (VKAs) in this setting, although the overall bleeding risk remains significant and evidence from randomized trials remains limited [59,60].

Similarly, patients with cancer undergoing PCI need dual antiplatelet therapy to prevent stent thrombosis, yet malignancy significantly increases bleeding risk. Recent data show that incorporating active cancer into bleeding risk scores such as PRECISE-DAPT substantially increases the proportion of patients classified as high bleeding risk [61]. This supports careful individualization and consideration of shorter dual antiplatelet therapy durations in selected oncologic patients. Despite the growing importance of cardio-oncology, patients with active malignancy remain markedly underrepresented in randomized antithrombotic trials. Consequently, current recommendations are largely extrapolated from non-cancer populations, limiting their applicability to this complex group [48,61,62,63].

In this setting, antithrombotic decisions should integrate not only conventional ischemic and bleeding risk, but also tumor type, disease stage, platelet count, expected need for surgery or biopsy, active chemotherapy, drug–drug interactions, renal or hepatic dysfunction, and the presence of mucosal or intracranial lesions. Patients with gastrointestinal, genitourinary, intracranial, metastatic, or hematologic malignancies, as well as those with thrombocytopenia or ongoing invasive procedures, should be considered at particularly high bleeding risk [53,54,55,56,64]. In cancer patients undergoing PCI, clopidogrel is generally the preferred P2Y12 receptor inhibitor when DAPT is required, whereas more potent drugs should be used cautiously because of bleeding concerns and limited evidence in this population [65]. When bleeding risk predominates and contemporary drug-eluting stents are used, abbreviated DAPT may be considered, with 1–3 months representing a reasonable strategy in selected oncologic patients, especially when active cancer therapy or invasive procedures cannot be delayed [64,65]. Conversely, in patients with ACS, complex PCI, prior stent thrombosis, or extensive coronary disease, longer DAPT may still be considered after multidisciplinary discussion. Reassessment should be repeated during active cancer treatment and whenever platelet count declines, bleeding occurs, chemotherapy changes, renal or hepatic function deteriorates, or surgery/biopsy is planned.

### 4.4. Atrial Fibrillation and Percutaneous Coronary Intervention

The coexistence of AF and recent PCI represents one of the most challenging scenarios in contemporary antithrombotic therapy. Oral anticoagulation is recommended for the prevention of cardioembolic stroke in patients with AF, and DAPT (aspirin plus a P2Y12 inhibitor) is recommended for preventing stent thrombosis and recurring coronary ischemic events following PCI and acute coronary syndrome. Patients with both indications therefore require antithrombotic regimens that combine oral anticoagulation and antiplatelet therapy. The therapeutic goal is to prevent both cardioembolic and coronary ischemic events while minimizing hemorrhagic risk [66].

Historically, triple therapy, consisting of oral anticoagulation (OAC), aspirin, and a P2Y12 inhibitor, was commonly used in patients with coexisting AF and recent coronary stenting, with the rationale of preventing cardioembolic stroke and stent thrombosis at the same time. Growing clinical evidence indicates that this intensive therapy increases bleeding complications, which have been independently associated with increased mortality and recurrent ischemic events, partly through interruption of antithrombotic therapy [1,2,6,7].

Over the past decade, several randomized trials have progressively challenged the paradigm of prolonged triple therapy. Early evidence from the WOEST trial demonstrated that the omission of aspirin in patients receiving OAC and clopidogrel significantly reduced bleeding without an apparent increase in thrombotic complications [67]. Similarly, the ISAR-TRIPLE trial showed no difference in ischemic outcomes between six weeks and six months of triple therapy, suggesting that prolonged exposure to triple therapy may not provide additional benefit while increasing hemorrhagic risk [68]. Subsequent trials evaluating DOAC-based strategies have further reshaped the therapeutic landscape. The PIONEER AF-PCI trial demonstrated that rivaroxaban-based regimens combined with a P2Y12 inhibitor reduced clinically significant bleeding compared with VKA-based triple therapy [69]. Consistent findings were observed in the RE-DUAL PCI trial, where dual therapy with dabigatran and a P2Y12 inhibitor significantly lowered bleeding events while maintaining comparable protection against thromboembolic outcomes [70]. Similarly, the AUGUSTUS trial showed that apixaban-based regimens were associated with lower bleeding rates compared with VKA-based therapy and that the omission of aspirin further reduced hemorrhagic events without a substantial increase in ischemic complications [71]. The ENTRUST-AF PCI trial subsequently confirmed that dual therapy with edoxaban plus a P2Y12 inhibitor was non-inferior to VKA-based triple therapy with respect to bleeding outcomes while preserving similar efficacy in preventing ischemic events [72].

Taken together, these trials consistently suggest that dual therapy combining a DOAC with a single antiplatelet agent—most commonly clopidogrel—reduces bleeding complications compared with traditional triple therapy, without a clear increase in major ischemic events in the overall trial populations. Consequently, contemporary guidelines increasingly favor simplified regimens and recommend minimizing the duration of triple therapy whenever possible [1,2,6,7,73].

In many patients, triple therapy is now limited to the periprocedural phase or to a very short course, followed by dual therapy with a DOAC and a P2Y12 inhibitor for several months and subsequently long-term anticoagulation alone. Importantly, these findings should not be interpreted as a universal abandonment of triple therapy. In selected patients with particularly high ischemic risk—such as those undergoing complex PCI or presenting with extensive coronary disease—a short course of triple therapy may still be clinically justified. In this context, the choice and duration of antithrombotic therapy require careful consideration of the individual balance between thrombotic and bleeding risk [74,75].

This issue becomes particularly relevant in the special populations that increasingly characterize contemporary cardiovascular practice. In elderly and frail patients, as well as in those with chronic kidney disease, active malignancy, or multiple comorbidities, the choice between triple therapy, dual therapy, or early treatment simplification cannot rely on trial-based algorithms alone. In these settings, both thrombotic and bleeding risks are often simultaneously amplified, while drug handling, treatment tolerance, and competing clinical priorities may substantially modify the net clinical benefit of antithrombotic therapy [76].

Therefore, the coexistence of AF and PCI should not be viewed merely as a question of how long triple therapy should be maintained, but rather as a paradigm of individualized antithrombotic decision-making in biologically vulnerable patients. This perspective is especially relevant in special populations, in whom renal dysfunction, frailty, cancer-related factors, polypharmacy, and procedural complexity frequently coexist and challenge the direct application of standard therapeutic strategies.

Operationally, most patients with AF undergoing PCI should receive early aspirin discontinuation followed by dual therapy with a DOAC and clopidogrel, when DOACs are not contraindicated [67,69,70,71,72,77]. Aspirin exposure should generally be restricted to the periprocedural phase or to the shortest clinically appropriate period, while a short course of triple therapy may still be justified in selected patients with high ischemic or stent-thrombotic risk, including STEMI presentation, complex PCI, left main or bifurcation stenting, multiple stents, prior stent thrombosis, or suboptimal procedural result [66,73,77,78]. Contemporary real-world data suggest that immediate double therapy may be safe in many patients with AF after PCI, but may carry a higher risk of stent thrombosis, particularly after STEMI, supporting careful selection of patients who may benefit from an initial short triple-therapy phase [78]. Clopidogrel should generally be preferred as the P2Y12 inhibitor combined with oral anticoagulation, whereas ticagrelor or prasugrel should be reserved for exceptional cases because of bleeding concerns and limited evidence in this setting.

After the initial combination phase, treatment should be stepped down to dual therapy and subsequently to long-term oral anticoagulation alone in clinically stable patients. In frail patients and in those with CKD or active cancer, treatment duration and drug selection should be reassessed according to renal function, hemoglobin, platelet count, bleeding symptoms, drug interactions, and changes in clinical status [72].

The main clinical considerations across the selected high-risk scenarios are summarized in Table 1, emphasizing both bleeding-sparing strategies and situations in which more intensive antithrombotic therapy may still be justified.

## 5. Limitations of Current Bleeding Risk Scores

Bleeding risk scores remain useful for structuring clinical assessment. However, their main limitation is that they convert a highly heterogeneous and dynamic clinical reality into simplified baseline classifications. Consequently, they are more effective at standardizing risk categorization than at guiding nuanced therapeutic decisions in complex patients [16,17,79]. Most existing models were developed to assess bleeding risk in specific patient populations over a defined time frame, based on a limited set of baseline data, and therefore provide a “snapshot” risk assessment. In clinical practice, however, bleeding risk in patients receiving antithrombotic therapy is dynamic and may be influenced by factors beyond baseline variables, including fluctuating renal function, active cancer therapy, new medications, procedural complexity, thrombocytopenia, anemia, and clinical instability. These dynamic modifiers, together with the limitations of available bleeding risk assessment models, can significantly alter hemorrhagic risk long after the initial therapeutic decision [80,81].

Importantly, the limitations of risk scores differ according to the tool considered. PRECISE-DAPT incorporates age, creatinine clearance, hemoglobin, white blood cell count, and previous bleeding, but it does not directly account for frailty, active cancer stage, thrombocytopenia, chemotherapy-related bleeding risk, or changes in renal function over time [82]. The DAPT score may help estimate the potential benefit of prolonged DAPT, but it was not designed to guide treatment in patients with marked biological vulnerability, active malignancy, severe CKD, or concomitant oral anticoagulation [83]. ARC-HBR provides a standardized and clinically useful definition of high bleeding risk, but it remains primarily a baseline classification and does not indicate how antithrombotic therapy should be modified when risk factors evolve after PCI [10]. The PARIS score integrates thrombotic and bleeding risk after PCI, but its practical applicability remains limited in frail, oncologic, or advanced CKD patients, particularly when treatment decisions must be repeatedly adapted over time [84]. Similarly, AF-related bleeding scores such as HAS-BLED, ORBIT, or ATRIA may support bleeding risk assessment in anticoagulated patients, but they do not adequately incorporate PCI complexity, stent-thrombotic risk, active cancer treatment, or the need for combined antiplatelet and anticoagulant therapy [85,86,87]. Finally, ACS-related tools such as CRUSADE or ACUITY are mainly oriented toward early or in-hospital bleeding risk and are less informative for long-term antithrombotic de-escalation in complex post-PCI patients [88,89].

A second concern is conceptual. Many current scores focus primarily on classical predictors of hemorrhage but fail to incorporate the underlying biological factors that may define vulnerability to therapy in contemporary practice. Elements such as frailty, functional impairment, non-cardiovascular comorbidities, treatment intolerance, and disease-specific vulnerabilities related to cancer or advanced CKD are, at best, inadequately represented [4,61,90,91]. From this perspective, future developments in hemorrhage risk prediction may depend more on incorporating disease-specific domains and repeated assessment over time than on further refining existing static models.

Frailty-oriented tools, cancer-adapted models, and renal function-integrated strategies may represent more clinically meaningful steps forward than the simple addition of further conventional variables to existing scores [49,61,92].

In parallel, artificial intelligence and machine learning-based models may help identify nonlinear interactions among comorbidities, treatments, and temporal changes that are difficult to capture through traditional risk scores alone [93,94,95].

However, the value of these newer approaches will ultimately depend on whether they improve clinical decision-making rather than prediction in isolation. The relevant question is not only whether a model estimates bleeding more accurately, but whether it helps clinicians tailor the intensity, combination, and duration of antithrombotic therapy in a way that improves net clinical benefit. Until such tools are prospectively validated and integrated into routine care, bleeding risk assessment should remain a dynamic clinical process supported—but not dictated—by formal scores.

The main characteristics and limitations of commonly used bleeding and ischemic risk tools are summarized in Table 2**,** with emphasis on their applicability to complex post-PCI patients.

## 6. Discussion and Future Directions

The clinical question this review aims to answer is not whether antithrombotic therapy remains a pillar of cardiovascular care, but how it should be interpreted and applied in patients whose clinical complexity extends beyond the populations used to develop current guidelines. Across the scenarios examined, a recurring pattern emerges: the patients in whom antithrombotic choices matter most are often those in whom the available evidence is least directly applicable.

Frailty, CKD, active malignancy, and atrial fibrillation requiring PCI are examples of settings where thrombotic and bleeding risk frequently coexist and change over time. Thus, treatment considerations should not end with selecting an initial standard strategy, but should also include treatment intensity and duration, timely de-escalation including aspirin discontinuation, the role and duration of triple therapy, and planned reassessment of risks and benefits. The limitations of static risk stratification are highlighted throughout this review. Bleeding risk scores are useful tools, but they cannot fully account for changes in renal function, platelet count, anemia, active cancer treatment, drug interactions, frailty progression, or procedural risk. Formal scores should therefore support clinical judgment, not replace it.

**Figure 1:**
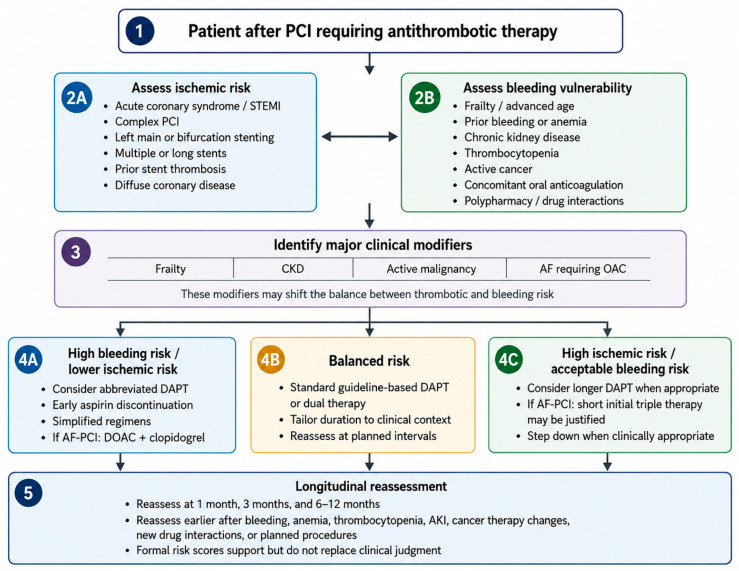
Practical framework for antithrombotic decision-making after PCI in high-risk cardiovascular patients. Abbreviations: AF, atrial fibrillation; AKI, acute kidney injuryCKD, chronic kidney disease; DAPT, dual antiplatelet therapy; DOAC, direct oral anticoagulant; OAC, oral anticoagulation; PCI, percutaneous coronary intervention; STEMI, ST-segment elevation myocardial infarction.

A practical framework integrating ischemic risk, bleeding vulnerability, patient-specific modifiers, and longitudinal reassessment is outlined in Figure 1. Future studies should enroll patients more representative of routine clinical practice, including frail elderly patients, individuals with advanced CKD or active malignancy, and patients with overlapping indications for antiplatelet and anticoagulant therapy.

Multidimensional and longitudinal risk models may help personalize treatment if they are prospectively validated and shown to improve clinical decision-making and patient outcomes. Ultimately, antithrombotic management after PCI in high-risk patients should be seen as an adaptive process. Rather than seeking a single universally optimal regimen, clinicians should repeatedly align treatment intensity, antithrombotic combinations, and duration with the patient’s evolving ischemic and bleeding risk.

## Figures and Tables

**Table 1:** Practical antithrombotic considerations in selected high-risk scenarios after PCI.

Clinical Scenario	Thrombotic Drivers	Bleeding Drivers	Evidence Gaps/Score Limitations	Practical Considerations
Frailty/older patients	Atherosclerotic burden; inflammation; immobility; ACS or complex CAD	Anemia; falls; renal dysfunction; polypharmacy; cognitive impairment	Frailty, falls, functional decline, and adherence are poorly captured by conventional scores [3,4,25,31]	Assess biological vulnerability beyond formal scores. If HBR and no major ischemic features, consider abbreviated DAPT or early de-escalation. Reassess after discharge and at 1–3 months [32,33,34].
Chronic kidney disease	Endothelial dysfunction; inflammation; platelet activation; vascular calcification; diffuse CAD	Uremic platelet dysfunction; anemia; drug accumulation; fluctuating renal function	Advanced CKD and dialysis patients are underrepresented in trials; static scores may not capture renal function changes over time [35,36,39,40]	Stratify by CKD severity and stability. In advanced CKD or dialysis, avoid prolonged combination therapy unless high ischemic or procedural risk is present. Use CrCl/eGFR for DOAC eligibility and dose adjustment; reassess after AKI, contrast exposure, hospitalization, or new interacting drugs [38,43,46,47].
Active cancer	Tumor-related coagulation; inflammation; endothelial injury; anticancer therapies; metastatic disease	Thrombocytopenia; mucosal lesions; GI/GU/intracranial tumor sites; invasive procedures	Cancer-specific risks and thrombocytopenia are incompletely represented in standard scores and PCI/DAPT trials [49,61,62,63,64,65]	Consider tumor site, stage, platelet count, chemotherapy, drug interactions, and planned procedures. Prefer clopidogrel if DAPT is required. In selected HBR patients, consider abbreviated DAPT, often 1–3 months, with repeated reassessment during active treatment [64,65].
AF undergoing PCI	Cardioembolism; stent thrombosis; ACS/STEMI; complex PCI; prior stent thrombosis	Combination therapy; advanced age; CKD; anemia; cancer; polypharmacy	AF-PCI trials support dual therapy but are underpowered for rare ischemic events and high stent-thrombotic-risk subgroups [67,68,69,70,71,72,78]	Default strategy: early aspirin discontinuation, then DOAC plus clopidogrel when eligible. Consider short triple therapy for STEMI, complex PCI, left main/bifurcation stenting, multiple stents, prior stent thrombosis, or suboptimal PCI result. Step down to OAC alone when stable [73,77,78].

Abbreviations. ACS, acute coronary syndrome; AF, atrial fibrillation; AKI, acute kidney injury; CAD, coronary artery disease; CKD, chronic kidney disease; CrCl, creatinine clearance; DAPT, dual antiplatelet therapy; DOAC, direct oral anticoagulant; eGFR, estimated glomerular filtration rate; GI, gastrointestinal; GU, genitourinary; HBR, high bleeding risk; OAC, oral anticoagulation; PCI, percutaneous coronary intervention; STEMI, ST-segment elevation myocardial infarction.

**Table 2:** Main bleeding and ischemic risk tools relevant to antithrombotic decision-making after PCI.

Score/Tool	Usual Setting	Main Value	Key Limitation
PRECISE-DAPT	DAPT after PCI	Estimates bleeding risk and may support DAPT duration	Does not capture frailty, active cancer features, thrombocytopenia, chemotherapy-related bleeding, or dynamic renal changes [82].
DAPT score	Prolonged DAPT after PCI	Estimates potential benefit of extending DAPT beyond 1 year	Less useful in biologically vulnerable patients, severe CKD, active malignancy, or concomitant OAC [83].
ARC-HBR criteria	HBR definition after PCI	Provides a standardized baseline HBR classification	Does not specify how to adapt treatment when bleeding risk changes over time [10].
PARIS score	Post-PCI ischemic and bleeding risk	Integrates thrombotic and bleeding risk after PCI	Limited in frail, oncologic, or advanced CKD patients needing repeated reassessment [84].
HAS-BLED/ORBIT/ATRIA	AF patients receiving anticoagulation	Supports bleeding risk assessment during OAC	Does not include PCI complexity, stent-thrombotic risk, cancer therapy, or combination antithrombotic treatment [85,86,87].
CRUSADE/ACUITY	ACS/invasive ACS management	Estimates early or in-hospital bleeding risk	Less useful for long-term de-escalation, DAPT duration, or AF-PCI decisions [88,89].

Abbreviations. ACS, acute coronary syndrome; AF, atrial fibrillation; ARC-HBR, Academic Research Consortium for High Bleeding Risk; CKD, chronic kidney disease; DAPT, dual antiplatelet therapy; HBR, high bleeding risk; OAC, oral anticoagulation; PCI, percutaneous coronary intervention.

## Data Availability

No new data were created or analyzed in this study. Data sharing is not applicable to this article.

## References

[1] Vrints C., Andreotti F., Koskinas K.C., Rossello X., Adamo M., Ainslie I., Banning A.P., Budaj A., Buechel R.R., Chieffo A. (2024). 2024 ESC Guidelines for the management of chronic coronary syndromes. Eur. Heart J..

[2] Byrne R.A., Rossello X., Coughlan J.J., Barbato E., Berry C., Chieffo A., Claeys M.J., Dweck M.R., Galbraith M., Gilard M. (2023). 2023 ESC Guidelines for the management of acute coronary syndromes. Eur. Heart J..

[3] Giallauria F., Di Lorenzo A., Venturini E., Pacileo M., D’Andrea A., Garofalo U., Delucia F., Testa C., Cuomo G., Vigorito C. (2021). Frailty in Acute and Chronic Coronary Syndrome Patients Entering Cardiac Rehabilitation. J. Clin. Med..

[4] Faridi K.F., Strom J.B., Kundi H., Butala N.M., Tamez H., Song Y., Shen C., Secemsky E.A., Yeh R.W., Curtis J.P. (2023). Association Between Claims-Defined Frailty and Outcomes Following 30 Versus 12 Months of Dual Antiplatelet Therapy After Percutaneous Coronary Intervention: Findings from the EXTEND-DAPT Study. J. Am. Heart Assoc..

[5] Damluji A.A., Forman D.E., Wang T.Y., Chikwe J., Kunadian V., Rich M.W., Young B.A., Page R.L., DeVon H.A., Alexander K.P. (2023). Management of Acute Coronary Syndrome in the Older Adult Population: A Scientific Statement from the American Heart Association. Circulation.

[6] Virani S.S., Newby L.K., Arnold S.V., Bittner V., Brewer L.C., Demeter S.H., Dixon D.L., Fearon W.F., Hess B., Johnson H.M. (2023). 2023 AHA/ACC/ACCP/ASPC/NLA/PCNA Guideline for the Management of Patients with Chronic Coronary Disease: A Report of the American Heart Association/American College of Cardiology Joint Committee on Clinical Practice Guidelines. Circulation.

[7] Rao S.V., O’Donoghue M.L., Ruel M., Rab T., Tamis-Holland J.E., Alexander J.H., Baber U., Baker H., Cohen M.G., Cruz-Ruiz M. (2025). 2025 ACC/AHA/ACEP/NAEMSP/SCAI guideline for the management of patients with acute coronary syndromes: A report of the American College of Cardiology/American Heart Association Joint Committee on Clinical Practice Guidelines. J. Am. Coll. Cardiol..

[8] Chang C.C., Ng A.K., Kogame N., Huang P.H., Kim B.K., van Geuns R.M. (2025). Decoding Bleeding Risks and Survival in Patients Undergoing Percutaneous Coronary Intervention on Antiplatelet Therapy. JACC Asia.

[9] Silverio A., Di Maio M., Buccheri S., De Luca G., Esposito L., Sarno G., Vecchione C., Galasso G. (2022). Validation of the academic research consortium high bleeding risk criteria in patients undergoing percutaneous coronary intervention: A systematic review and meta-analysis of 10 studies and 67,862 patients. Int. J. Cardiol..

[10] Urban P., Mehran R., Colleran R., Angiolillo D.J., Byrne R.A., Capodanno D., Cuisset T., Cutlip D., Eerdmans P., Eikelboom J. (2019). Defining High Bleeding Risk in Patients Undergoing Percutaneous Coronary Intervention. Circulation.

[11] Rodriguez Lozano P., Bourque J.M. (2022). Beyond traditional cardiovascular risk factors: Could frailty and other morbidities explain the worse prognosis in patients undergoing pharmacologic stress?. J. Nucl. Cardiol..

[12] Perone F., Bernardi M., Spadafora L., Betti M., Cacciatore S., Saia F., Fogacci F., Jaiswal V., Asher E., Paneni F. (2025). Non-Traditional Cardiovascular Risk Factors: Tailored Assessment and Clinical Implications. J. Cardiovasc. Dev. Dis..

[13] Valgimigli M., Frigoli E., Heg D., Tijssen J., Jüni P., Vranckx P., Ozaki Y., Morice M.C., Chevalier B., Onuma Y. (2021). Dual Antiplatelet Therapy after PCI in Patients at High Bleeding Risk. N. Engl. J. Med..

[14] Farrokh S., Nalleballe K., Onteddu S., Suarez J.I., Bösel J., Shah V.A. (2025). Bleeding Risk with Combining Antiplatelets and Anticoagulants for Secondary Stroke Prevention: A Propensity Score-Matched Analysis. J. Am. Heart Assoc..

[15] Galanti K., Di Marino M., Mansour D., Testa S., Rossi D., Scollo C., Magnano R., Pezzi L., D’Alleva A., Forlani D. (2024). Current Antithrombotic Treatments for Cardiovascular Diseases: A Comprehensive Review. Rev. Cardiovasc. Med..

[16] Bauer D., Fojtík A., Berka V., Neuberg M., Kočka V., Prachtová R., Toušek P. (2026). Prognostic Role of the PRECISE-DAPT Score in Acute Coronary Syndrome and Different Antithrombotic Treatment Strategies. Cardiol. Ther..

[17] Singh A., Hussain M.A., Chaudhary S.C., Bharadwaj A., Sawalani K.K., Pradhan A., Sethi R. (2024). Assessing the Utility of the DAPT Score and PRECISE-DAPT Score in Determining the Appropriateness of Dual Antiplatelet Therapy in Patients With Acute Myocardial Infarction/Percutaneous Coronary Intervention. Cardiol. Res. Pract..

[18] Ueki Y., Bär S., Losdat S., Otsuka T., Zanchin C., Zanchin T., Gragnano F., Gargiulo G., Siontis G.C.M., Praz F. (2020). Validation of the Academic Research Consortium for High Bleeding Risk (ARC-HBR) criteria in patients undergoing percutaneous coronary intervention and comparison with contemporary bleeding risk scores. EuroIntervention.

[19] Marschall A.F., Duarte Torres J., Biscotti Rodíl B., Gómez Sánchez I., Basabe Velasco E., Ramos Alejos-Pita C., López Soberón E., Suárez Cuervo A., Álvarez Antón S., de la Torre Hernández J.M. (2024). PRECISE-DAPT, ARC-HBR, or Simplified Clinical Evaluation for the Prediction of Major Bleeding After Percutaneous Coronary Intervention in older Patients. Am. J. Cardiol..

[20] Ko S.Q., Valsdottir L.R., Strom J.B., Cheng Y.C., Hirayama A., Liu P.H., Yanagisawa N., Yen H., Shen C., Yeh R.W. (2018). Meta-Analysis of Bleeding Risk Prediction Scores in Patients After Percutaneous Coronary Intervention on Dual Antiplatelet Therapy. Am. J. Cardiol..

[21] Munafò A.R., Montalto C., Franzino M., Pistelli L., Di Bella G., Ferlini M., Leonardi S., D’Ascenzo F., Gragnano F., Oreglia J.A. (2023). External validity of the PRECISE-DAPT score in patients undergoing PCI: A systematic review and meta-analysis. Eur. Heart J. Cardiovasc. Pharmacother..

[22] Thiruchelvam K., Chun Xin J.T., Law W.K., Lee L.F., Liew X.B., Le Lim J., Hui Min O.S., Tan Z.Q., Kow C.S. (2025). Bleeding risk assessment tools for patients with myocardial infarction: A comparative review and clinical implications. Expert Rev. Cardiovasc. Ther..

[23] Price M.J. (2020). Abbreviated Dual Antiplatelet Therapy After Percutaneous Coronary Intervention in High Bleeding Risk Patients: LEADERS-FREE and ONYX ONE. Interv. Cardiol. Clin..

[24] Landi A., Gorog D.A. (2026). Optimizing risks and benefits of dual antiplatelet therapy after ACS: A winding path to precision. Future Cardiol..

[25] Clegg A., Young J., Iliffe S., Rikkert M.O., Rockwood K. (2013). Frailty in elderly people. Lancet.

[26] Doody P., Lord J.M., Greig C.A., Whittaker A.C. (2023). Frailty: Pathophysiology, Theoretical and Operational Definition(s), Impact, Prevalence, Management and Prevention, in an Increasingly Economically Developed and Ageing World. Gerontology.

[27] Saint Croix G., Lacy S.C., Gazzhal A., Ibrahim M., Gjergjindreaj M., Perez J., Shehadeh M., Vedantam K., Torres C., Beohar N. (2022). Dual Antiplatelet Therapy in Patients Aged 75 Years and Older with Coronary Artery Disease: A Meta-Analysis and Systematic Review. J. Interv. Cardiol..

[28] Mohebali D., Kaplan D., Carlisle M., Supiano M.A., Rondina M.T. (2014). Alterations in platelet function during aging: Clinical correlations with thromboinflammatory disease in older adults. J. Am. Geriatr. Soc..

[29] Sciahbasi A., Minardi S., Salvi N., Infusino F., Granatelli A. (2025). Antithrombotic Therapy in the Elderly with Cardiovascular Disease: Walking the Tightrope Between Efficacy and Bleeding Risk-A Narrative Review. J. Clin. Med..

[30] Shimono H., Tokushige A., Kanda D., Ohno A., Hayashi M., Fukuyado M., Akao M., Kawasoe M., Arikawa R., Otsuji H. (2023). Association of preoperative clinical frailty and clinical outcomes in elderly patients with stable coronary artery disease after percutaneous coronary intervention. Heart Vessel..

[31] Dodson J.A., Hochman J.S., Roe M.T., Chen A.Y., Chaudhry S.I., Katz S., Zhong H., Radford M.J., Udell J.A., Bagai A. (2018). The Association of Frailty with In-Hospital Bleeding Among Older Adults with Acute Myocardial Infarction: Insights from the ACTION Registry. JACC Cardiovasc. Interv..

[32] Pavasini R., Maietti E., Tonet E., Bugani G., Tebaldi M., Biscaglia S., Cimaglia P., Serenelli M., Ruggiero R., Vitali F. (2019). Bleeding Risk Scores and Scales of Frailty for the Prediction of Haemorrhagic Events in Older Adults with Acute Coronary Syndrome: Insights from the FRASER study. Cardiovasc. Drugs Ther..

[33] Qian Y., Xu B., Qian X., Cao L., Cheng Y., Liu X., Bai S., Han Z., Wang J. (2021). Incidence and Risk Factors for Antiplatelet Therapy-Related Bleeding Complications Among Elderly Patients After Coronary Stenting: A Multicenter Retrospective Observation. Front. Pharmacol..

[34] Park D.Y., Hu J.R., Jamil Y., Kelsey M.D., Jones W.S., Frampton J., Kochar A., Aronow W.S., Damluji A.A., Nanna M.G. (2024). Shorter Dual Antiplatelet Therapy for Older Adults After Percutaneous Coronary Intervention: A Systematic Review and Network Meta-Analysis. JAMA Netw. Open.

[35] Saeed Z., Sirolli V., Bonomini M., Gallina S., Renda G. (2024). Hallmarks for Thrombotic and Hemorrhagic Risks in Chronic Kidney Disease Patients. Int. J. Mol. Sci..

[36] Di Mauro M., Fiorentini V., Mistrulli R., Veneziano F.A., De Luca L. (2022). Acute coronary syndrome and renal impairment: A systematic review. Rev. Cardiovasc. Med..

[37] Baaten C.C.F.M.J., Schröer J.R., Floege J., Marx N., Jankowski J., Berger M., Noels H. (2022). Platelet Abnormalities in CKD and Their Implications for Antiplatelet Therapy. Clin. J. Am. Soc. Nephrol..

[38] Aursulesei V., Costache I.I. (2019). Anticoagulation in chronic kidney disease: From guidelines to clinical practice. Clin. Cardiol..

[39] Haysom R., Aziz N., Swan D., Thachil J. (2025). Bleeding in Patients with Renal Impairment: A Current Perspective. Semin. Thromb. Hemost..

[40] Mahady S.E., Polekhina G., Woods R.L., Wolfe R., Wetmore J.B., Margolis K.L., Wood E.M., Cloud G.C., Murray A.M., Polkinghorne K.R. (2023). Association Between CKD and Major Hemorrhage in Older Persons: Data from the Aspirin in Reducing Events in the Elderly Randomized Trial. Kidney Int. Rep..

[41] Düsing P., Zietzer A., Goody P.R., Hosen M.R., Kurts C., Nickenig G., Jansen F. (2021). Vascular pathologies in chronic kidney disease: Pathophysiological mechanisms and novel therapeutic approaches. J. Mol. Med..

[42] Ozyuncu N. (2025). Antiplatelet Therapy in Heart Disease. Rev. Cardiovasc. Med..

[43] Parul F., Ratnani T., Subramani S., Bhatia H., Ashmawy R.E., Nair N., Manchanda K., Anyagwa O.E., Kaka N., Patel N. (2025). Anticoagulation in Patients with End-Stage Renal Disease: A Critical Review. Healthcare.

[44] Natale P., Palmer S.C., Saglimbene V.M., Ruospo M., Razavian M., Craig J.C., Jardine M.J., Webster A.C., Strippoli G.F.M. (2022). Antiplatelet agents for chronic kidney disease. Cochrane Database Syst. Rev..

[45] Israr M.M., Aftab T., Sainani P., Zuberi R., Fredie B.L., Tayyab M., Ur Rehman A., Nadeem S. (2025). Prevalence of Chronic Myocardial Ischemia and Gastrointestinal Bleeding Risk in Patients with Chronic Kidney Disease Undergoing Dual Antiplatelet Therapy. Cureus.

[46] Gutiérrez O.M. (2019). Risks of anticoagulation in patients with chronic kidney disease and atrial fibrillation: More than just bleeding?. Res. Pract. Thromb. Haemost..

[47] Calderon J.M., Martinez F., Fernandez A., Sauri I., Diaz J., Uso R., Trillo J.L., Redon J., Forner M.J. (2022). Real world data of anticoagulant treatment in non-valvular atrial fibrillation across renal function status. Sci. Rep..

[48] Cereda A., Lucreziotti S., Franchina A.G., Laricchia A., De Regibus V., Conconi B., Carlà M., Spangaro A., Rocchetti M., Ponti L. (2023). Systematic Review and Meta-Analysis of Oral Anticoagulant Therapy in Atrial Fibrillation Cancer Patients. Cancers.

[49] Dafaalla M., Costa F., Kontopantelis E., Araya M., Kinnaird T., Micari A., Jia H., Mintz G.S., Mamas M.A. (2024). Bleeding risk prediction after acute myocardial infarction-integrating cancer data: The updated PRECISE-DAPT cancer score. Eur. Heart J..

[50] Poénou G., Tolédano E., Helfer H., Plaisance L., Happe F., Versini E., Diab N., Djennaoui S., Mahé I. (2023). Assessment of bleeding risk in cancer patients treated with anticoagulants for venous thromboembolic events. Front. Cardiovasc. Med..

[51] Roy D.C., Wang T.F., Mallick R., Burger D., Carrier M., Wells P., Hawken S. (2026). Venous thromboembolism and bleeding in cancer patients: Role of inflammatory and cardiac biomarkers. Eur. Heart J..

[52] Alshehri F.S., Alloghbi A., Alshalani A., Sabah H.A., Whyte C.S. (2025). Cancer-Associated Thrombosis (CAT); mechanisms and treatment options. Thromb. J..

[53] Ilari A., Abbate M.I., Verso M., Graziani M., Cafaro P., Sala L., Colonese F., Cortinovis D.L., Canova S. (2026). Cancer-Associated Thrombosis in Patients Treated with Immune Checkpoint Inhibitors. Int. J. Mol. Sci..

[54] Vedovati M.C., Talerico R., Sacco C., Mazzetti M., Campello E., Birocchi S., Beccatini S., Porfidia A., Caprari R., Lodigiani C. (2026). Major Bleeding Risk Assessment in Patients with Cancer-Associated Venous Thromboembolism Treated with DOACs: Data from a Multicenter Cohort. Thromb. Haemost..

[55] Loncharich A., Gage B.F., Luo S., Schoen M., Afzal A., Mahmoud A., Carson K., Chang S.-H., Yan Y., Sanfilippo K.M. (2025). Risk factors for anticoagulant-related bleeding in cancer: Traditional and cancer-specific factors. Blood Vessel. Thromb. Hemost..

[56] Escobar A., Salem A.M., Dickson K., Johnson T.N., Burk K.J., Bashoura L., Faiz S.A. (2022). Anticoagulation and bleeding in the cancer patient. Support. Care Cancer.

[57] Li Y., Jiang H., Luo L., Mei H. (2025). Immunotherapy-associated hemostatic abnormalities: Bleeding and thrombotic complications. Ann. Hematol..

[58] Wan T., Song J., Zhu D. (2025). Cancer-associated venous thromboembolism: A comprehensive review. Thromb. J..

[59] Mariani M.V., Magnocavallo M., Straito M., Piro A., Severino P., Iannucci G., Chimenti C., Mancone M., Della Rocca D.G., Forleo G.B. (2021). Direct oral anticoagulants versus vitamin K antagonists in patients with atrial fibrillation and cancer a meta-analysis. J. Thromb. Thrombolysis.

[60] Giustozzi M., Franco L., Agnelli G., Verso M. (2023). Unmet clinical needs in the prevention and treatment of cancer-associated venous thromboembolism. Trends Cardiovasc. Med..

[61] von Koch S., Mamas M.A., Dafaalla M., Costa F., Koul S., Jernberg T., Erlinge D., Mohammad M.A. (2025). Prediction of major bleeding events for patients with dual antiplatelet therapy after myocardial infarction-a validation of the PRECISE-DAPT cancer score. Eur. Heart J. Open.

[62] Campos C.M., Mehran R., Capodanno D., Owen R., Windecker S., Varenne O., Stone G.W., Valgimigli M., Hajjar L.A., Kalil Filho R. (2024). Risk Burden of Cancer in Patients Treated with Abbreviated Dual Antiplatelet Therapy After PCI: Analysis of Multicenter Controlled High-Bleeding Risk Trials. Circ. Cardiovasc. Interv..

[63] Guo W., Fan X., Lewis B.R., Johnson M.P., Rihal C.S., Lerman A., Herrmann J. (2021). Cancer Patients Have a Higher Risk of Thrombotic and Ischemic Events After Percutaneous Coronary Intervention. JACC Cardiovasc. Interv..

[64] Tufano A., Coppola A. (2024). How to manage anticoagulation for cancer-associated thrombosis and atrial fibrillation in cancer. Thromb. Update.

[65] Tsigkas G., Vakka A., Apostolos A., Bousoula E., Vythoulkas-Biotis N., Koufou E.-E., Vasilagkos G., Tsiafoutis I., Hamilos M., Aminian A. (2023). Dual Antiplatelet Therapy and Cancer; Balancing between Ischemic and Bleeding Risk: A Narrative Review. J. Cardiovasc. Dev. Dis..

[66] De Caterina R., Agewall S., Andreotti F., Angiolillo D.J., Bhatt D.L., Byrne R.A., Collet J.-P., Eikelboom J., Fanaroff A.C., Gibson C.M. (2022). Great debate: Triple antithrombotic therapy in patients with atrial fibrillation undergoing coronary stenting should be limited to 1 week. Eur. Heart J..

[67] Dewilde W.J., Oirbans T., Verheugt F.W.A., Kelder J.C., De Smet B.J.G.L., Herrman J.-P., Adriaenssens T., Vrolix M., Heestermans A.A.C.M., Vis M.M. (2013). Use of clopidogrel with or without aspirin in patients taking oral anticoagulant therapy and undergoing percutaneous coronary intervention: An open-label, randomised, controlled trial. Lancet.

[68] Fiedler K.A., Oirbans T., Verheugt F.W.A., Kelder J.C., De Smet B.J.G.L., Herrman J.-P., Adriaenssens T., Vrolix M., Heestermans A.A.C.M., Vis M.M. (2015). Duration of Triple Therapy in Patients Requiring Oral Anticoagulation After Drug-Eluting Stent Implantation: The ISAR-TRIPLE Trial. J. Am. Coll. Cardiol..

[69] Gibson C.M., Mehran R., Bode C., Halperin J., Verheugt F.W., Wildgoose P., Birmingham M., Ianus J., Burton P., van Eickels M. (2016). Prevention of bleeding in patients with atrial fibrillation undergoing PCI. N. Engl. J. Med..

[70] Cannon C.P., Bhatt D.L., Oldgren J., Lip G.Y.H., Ellis S.G., Kimura T., Maeng M., Merkely B., Zeymer U., Gropper S. (2017). Dual antithrombotic therapy with dabigatran after PCI in atrial fibrillation. N. Engl. J. Med..

[71] Lopes R.D., Heizer G., Aronson R., Vora A.N., Massaro T., Mehran R., Goodman S.G., Windecker S., Darius H., Li J. (2019). Antithrombotic Therapy after Acute Coronary Syndrome or PCI in Atrial Fibrillation. N. Engl. J. Med..

[72] Vranckx P., Valgimigli M., Eckardt L., Tijssen J., Lewalter T., Gargiulo G., Batushkin V., Campo G., Lysak Z., Vakaliuk I. (2019). Edoxaban-based versus vitamin K antagonist-based antithrombotic regimen after successful coronary stenting in patients with atrial fibrillation (ENTRUST-AF PCI): A randomised, open-label, phase 3b trial. Lancet.

[73] Andreotti F., Geisler T., Collet J.P., Gigante B., Gorog D.A., Halvorsen S., Lip G.Y.H., Morais J., Navarese E.P., Patrono C. (2023). Acute, periprocedural and long-term antithrombotic therapy in older adults: 2022 update by the ESC Working Group on Thrombosis. Eur. Heart J..

[74] Lutz J., Jurk K., Schinzel H. (2017). Direct oral anticoagulants in patients with chronic kidney disease: Patient selection and special considerations. Int. J. Nephrol. Renov. Dis..

[75] Kessler A., Kolben Y., Puris G., Ellis M., Alperin M., Simovich V., Lerman Shivek H., Muszkat M., Maaravi Y., Biton Y. (2023). Direct Oral Anticoagulants in Special Patient Populations. J. Clin. Med..

[76] Nusrat S., Khan S., Beg K., Raskob G. (2026). Unmet Needs and Challenges in Cancer-Associated Venous Thromboembolism. Int. J. Mol. Sci..

[77] Ziser K., Rahman S., Soro R., Falconer N., Harrop D. (2025). The role of triple antithrombotic therapy in patients with atrial fibrillation and coronary stent insertion. Aust. Prescr..

[78] Park D.Y., McLean B., Akman Z., Li D.K., Babapour G., Nanna M.G. (2025). Contemporary use and clinical significance of initial triple versus double therapy after percutaneous coronary intervention for myocardial infarction in patients with atrial fibrillation. J. Am. Heart Assoc..

[79] Lim C.E., Simonsson M., Pasternak B., Jernberg T., Edgren G., Ueda P. (2025). Discordance and Performance of the ARC-HBR and PRECISE-DAPT High Bleeding Risk Definitions After Coronary Stenting. JACC Cardiovasc. Interv..

[80] Englisch C., Vladic N., Ay C. (2025). Bleeding Risk in Patients with Cancer. Hamostaseologie.

[81] Sen L., Kangpin X., Yihui L. (2025). Research progress on bleeding risk assessment models in anticoagulant therapy. Front. Cardiovasc. Med..

[82] Costa F., van Klaveren D., James S., Heg D., Räber L., Feres F., Pilgrim T., Hong M.K., Kim H.S., Colombo A. (2017). Derivation and validation of the predicting bleeding complications in patients undergoing stent implantation and subsequent dual antiplatelet therapy (PRECISE-DAPT) score: A pooled analysis of individual-patient datasets from clinical trials. Lancet.

[83] Yeh R.W., Secemsky E.A., Kereiakes D.J., Normand S.L., Gershlick A.H., Cohen D.J., Spertus J.A., Steg P.G., Cutlip D.E., Rinaldi M.J. (2016). Development and validation of a prediction rule for benefit and harm of dual antiplatelet therapy beyond 1 year after percutaneous coronary intervention. JAMA.

[84] Baber U., Mehran R., Giustino G., Cohen D.J., Henry T.D., Sartori S., Ariti C., Litherland C., Dangas G., Gibson C.M. (2016). Coronary thrombosis and major bleeding after PCI with drug-eluting stents: Risk scores from PARIS. J. Am. Coll. Cardiol..

[85] Pisters R., Lane D.A., Nieuwlaat R., de Vos C.B., Crijns H.J., Lip G.Y. (2010). A novel user-friendly score (HAS-BLED) to assess 1-year risk of major bleeding in patients with atrial fibrillation: The Euro Heart Survey. Chest.

[86] O’Brien E.C., Simon D.N., Thomas L.E., Hylek E.M., Gersh B.J., Ansell J.E., Kowey P.R., Mahaffey K.W., Halperin J.L., Hankey G.J. (2015). The ORBIT bleeding score: A simple bedside score to assess bleeding risk in atrial fibrillation. Eur. Heart J..

[87] Fang M.C., Go A.S., Chang Y., Borowsky L.H., Pomernacki N.K., Udaltsova N., Singer D.E. (2011). A new risk scheme to predict warfarin-associated hemorrhage: The ATRIA (Anticoagulation and Risk Factors in Atrial Fibrillation) Study. J. Am. Coll. Cardiol..

[88] Subherwal S., Bach R.G., Chen A.Y., Gage B.F., Rao S.V., Newby L.K., Wang T.Y., Gibler W.B., Ohman E.M., Roe M.T. (2009). Baseline risk of major bleeding in non-ST-segment-elevation myocardial infarction: The CRUSADE Bleeding Score. Circulation.

[89] Mehran R., Pocock S.J., Nikolsky E., Clayton T., Dangas G.D., Kirtane A.J., Parise H., Fahy M., Manoukian S.V., Feit F. (2010). A risk score to predict bleeding in patients with acute coronary syndromes. J. Am. Coll. Cardiol..

[90] Alonso Salinas G.L., Sanmartín Fernández M., Pascual Izco M., Marco Del Castillo Á., Rincón Díaz L.M., Lozano Granero C., Valverde Gómez M., Pastor Pueyo P., Del Val Martín D., Pardo Sanz A. (2016). Frailty predicts major bleeding within 30 days in elderly patients with Acute Coronary Syndrome. Int. J. Cardiol..

[91] Montalto C., Crimi G., Morici N., Montalto C., Crimi G., Morici N., Piatti L., Grosseto D., Sganzerla P., Tortorella G. (2021). Bleeding risk prediction in elderly patients managed invasively for acute coronary syndromes: External validation of the PRECISE-DAPT and PARIS scores. Int. J. Cardiol..

[92] Dong L., Lu C., Wensen C., Fuzhong C., Khalid M., Xiaoyu D., Guangjuan L., Yanxia Q., Yufeng Z., Xinjian L. (2022). Performance of PRECISE-DAPT and Age-Bleeding-Organ Dysfunction Score for Predicting Bleeding Complication During Dual Antiplatelet Therapy in Chinese Elderly Patients. Front. Cardiovasc. Med..

[93] Yang Y., Yan Y., Zhou Z., Zhang J., Han H., Zhang W., Wang X., Chen C., Ge W., Pan J. (2025). Accurate prediction of bleeding risk after coronary artery bypass grafting with dual antiplatelet therapy: A machine learning model vs. the PRECISE-DAPT score. Int. J. Cardiol..

[94] Li F., Rasmy L., Xiang Y., Feng J., Abdelhameed A., Hu X., Sun Z., Aguilar D., Dhoble A., Du J. (2024). Dynamic Prognosis Prediction for Patients on DAPT After Drug-Eluting Stent Implantation: Model Development and Validation. J. Am. Heart Assoc..

[95] Qian Y., Wanlin L., Maofeng W. (2024). Machine learning derived model for the prediction of bleeding in dual antiplatelet therapy patients. Front. Cardiovasc. Med..

